# Psychometric evaluation of a visual analog scale for the assessment of anxiety

**DOI:** 10.1186/1477-7525-8-57

**Published:** 2010-06-08

**Authors:** Valerie SL Williams, Robert J Morlock, Douglas Feltner

**Affiliations:** 1 RTI Health Solutions, 3040 Cornwallis Road, Research Triangle Park NC 27707 USA; 2 Innovus, 12125 Technology Drive, Eden Prairie, MN 55344 USA; 3 Pfizer Inc., Eastern Point Road, Mailstop 8220-4301, Groton CT 06340 USA

## Abstract

**Background:**

Fast-acting medications for the management of anxiety are important to patients and society. Measuring early onset, however, requires a sensitive and clinically responsive tool. This study evaluates the psychometric properties of a patient-reported Global Anxiety - Visual Analog Scale (GA-VAS).

**Methods:**

Data from a double-blind, randomized, placebo-controlled study of lorazepam and paroxetine in patients with Generalized Anxiety Disorder were analyzed to assess the reliability, validity, responsiveness, and utility of the GA-VAS. The GA-VAS was completed at clinic visits and at home during the first week of treatment. Targeted psychometric analyses—test-retest reliabilities, validity correlations, responsiveness statistics, and minimum important differences—were conducted.

**Results:**

The GA-VAS correlates well with other anxiety measures, at Week 4, *r *= 0.60 (*p *< 0.0001) with the Hamilton Rating Scale for Anxiety and *r *= 0.74 (*p *< 0.0001) with the Hospital Anxiety and Depression Scale - Anxiety subscale. In terms of convergent and divergent validity, the GA-VAS correlated -0.54 (*p *< 0.0001), -0.48 (*p *< 0.0001), and -0.68 (*p *< 0.0001) with the SF-36 Emotional Role, Social Function, and Mental Health subscales, respectively, but correlated much lower with the SF-36 physical functioning subscales. Preliminary minimum important difference estimates cluster between 10 and 15 mm.

**Conclusions:**

The GA-VAS is capable of validly and effectively capturing a reduction in anxiety as quickly as 24 hours post-dose.

## 

Single-item visual analog scales (VASs) have been used in psychological assessment since the early 20th century and have subsequently been employed successfully in the assessment of a wide variety of health-related constructs including pain [1-3], quality-of-life [4,5], and mood [6-8]. VASs are brief and simple to administer and minimal in terms of respondent burden. These characteristics make them ideal for use in a diary format questionnaire where patients frequently record symptoms and outcomes.

VASs are particularly useful when assessing a single construct with many perceptible gradations and research has shown that unipolar VASs ("Not at all Anxious" to "Extremely Anxious") are more easily understood than bipolar VASs ("Extremely Calm" to "Extremely Anxious") [9]. Although a VAS may be oriented vertically, the most common form is a horizontal line. In fact, horizontal scales have been shown to produce a more uniform distribution of scores and to be more sensitive than vertical scales [3,10]. Multiple-item VASs are often shown to have high internal consistency [10]; however, there is wide variability in test-retest reliabilities—VAS test-retest reliability is generally not uniform across the scale continuum, but better at the middle and extremes [11]. Although VAS differences appear larger than differences on 7-point ordinal response items, when standardized there is generally no difference between VAS and ordinal ratings [12]. Similarly, VAS standard errors of measurement are proportionally larger than those for rating scales [12].

The present study was motivated by the need for a brief validated measure for assessing onset of improvement in the symptom of anxiety in subjects with GAD sooner than one week, especially in light of the need to evaluate newer fast-acting medications for the management of anxiety. The Hamilton Rating Scale for Anxiety (HAM-A) [13] is considered the "gold standard" and commonly used in clinical trials to assess response to anxiolytic treatment in patients with GAD. However, three important disadvantages of the HAM-A are that it is relatively lengthy (14 items), it must be completed by a trained clinician during the course of a clinical interview, and it has not been validated for use sooner than one week. In the context of GAD, treatment with fast-acting benzodiazepines has been shown to be effective at one week when measured by the HAM-A [14,15]; in the context of panic disorder [16] and anticipatory anxiety [17,18], self-report VASs and other measures have demonstrated efficacy within hours. As there are no validated single-item scales to assess onset of anxiety relief in patients with GAD, it seemed important to us to validate a VAS assessing average anxiety (over the past 24 hours), which could be easily incorporated into a daily diary. The present study describes the psychometric evaluation of a patient-reported VAS for the daily assessment of anxiety when used in a clinical trial assessing pharmaceutical treatments for GAD.

## Methods

### Preliminary Qualitative Study

As part of a preliminary qualitative study, cognitive interviews were conducted with 22 GAD patients (77.3% female), ranging in age from 21 to 59, to better understand the interpretation of the GA-VAS and the GA-VAS response process from the patient perspective. Patients were asked to think aloud while completing the GA-VAS so that the interviewer could hear how it was interpreted and how a response was selected.

### Psychometric Study Design

After cognitive testing, the GA-VAS was included in a clinical trial assessing two approved pharmaceutical treatments for anxiety. Analyses were aimed at providing evidence of the reliability, responsiveness, validity, and utility of the GA-VAS.

Data were collected during a randomized, 4-week, double-blind, multi-center, fixed-dose, placebo-controlled, parallel-group clinical study conducted in the United States. Lorazepam was selected as a fast-acting benzodiazepine, and paroxetine, a selective serotonin reuptake inhibitor, was chosen as a slower-acting GAD pharmacotherapy. There were three treatment arms—lorazepam (1.5 mg TID), paroxetine (20 mg QD), and placebo—and three phases to the study: (1) a 1-week screening phase (Days -7 to -1) during which eligibility was determined; (2) a 4-week double-blind treatment phase (Day 1 or baseline through Week 4); and (3) a 5-day double-blind treatment phase (Week 5) during which therapy was down-titrated. Patients completed the GA-VAS during six clinic visits (screening, baseline, Weeks 1, 2, 4, and 5) and at home each night during the screening week and first week of treatment.

#### Participants

Otherwise healthy individuals, aged 18 to 65 with a primary diagnosis of GAD as determined by a structured clinical interview, and a HAM-A total score ≥ 20 were eligible for inclusion. To ensure prominence of anxiety symptoms over depression symptoms, patients were required to have a Covi Anxiety Scale [19] score ≥ 9 and a Raskin Depression Scale [20] score ≤ 7. These psychiatric rating scales have long been used in clinical trials and have been shown to be valid tools for differentiating anxious and depressed patient subgroups [21,22]. Subjects were excluded from study participation if they had significant suicidal risk, had failed treatment with lorazepam or paroxetine in the past, required daily benzodiazepine use in the three months prior to study participation, or if they had most other concurrent DSM-IV mental disorders, including major depressive disorder, panic disorder with or without agoraphobia, acute stress disorder, obsessive compulsive disorder, dissociative disorder, posttraumatic stress disorder, social anxiety disorder, anorexia, bulimia, caffeine-induced anxiety disorder, alcohol or substance abuse or dependence, premenstrual dysphoric disorder, or antisocial or borderline personality disorder. Subjects with current or past diagnoses of schizophrenia, psychotic disorders, delirium, dementia, amnestic disorders, clinically significant cognitive disorders, bipolar or schizoaffective disorder, benzodiazepine abuse or dependence, or factitious disorder were also excluded. Patients were not permitted to use any psychotropic medications and could not have initiated any psychodynamic or behavioral psychotherapy for anxiety within the 3 months prior to the study.

#### Instruments

#### General Anxiety - Visual Analog Scale

**Figure 1:**
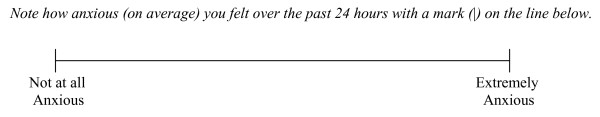
**The GA-VAS**. Please complete this form at a regular time each day, preferably just before going to bed, and consider the whole of the previous 24-hour period.

The 100 mm GA-VAS, shown in Figure 1 (not to scale), was administered at all clinic visits and at home in a daily diary format. The distance from the left edge of the line to the mark placed by the patient is measured to the nearest millimeter and used in analyses as the patient GA-VAS score.

A number of additional measures were included in the present psychometric evaluation study to help assess the construct validity of the GA-VAS. Both the HAM-A [13] and the Hospital Anxiety and Depression Scale (HADS) [23,24] were completed during clinic visits. The HAM-A is a clinician-reported measure of 14 items assessing both psychic or cognitive (anxious mood, fears, intellectual impairment, etc.) and somatic or physical symptoms of anxiety (muscular complaints, cardiovascular symptoms, gastrointestinal symptoms, etc.) on a 5-point severity scale (0 = "Not present" to 4 = "Very severe"). The HADS is a 14-item self-report measure designed to screen for mood disorders in medically ill patients. Seven HADS items assess anxiety and seven assess depression on a 0-to-3 response scale; anxiety and depression are scored separately. Like the GA-VAS, higher scores on the HAM-A and HADS reflect greater severity.

Two self-report instruments gathered generic information about patient quality-of-life, the 36-item Medical Outcomes Study Short Form - 36 (SF-36) [25,26] and the 14-item General Activity subscale of the Quality of Life Enjoyment and Satisfaction Questionnaire (QLES-Q) [27]. For each item of the QLES-Q, the respondent uses a 5-point scale ranging from 1 = "Very poor" satisfaction to 5 = "Very good" satisfaction; higher scores indicate greater quality-of-life and satisfaction. The SF-36 assesses eight dimensions of health-related functioning and quality-of-life: Physical Functioning, Physical Role, Bodily Pain, Social Functioning, General Mental Health, Emotional Role, Vitality, and General Health Perceptions. Each subscale is scored from 0 to 100, with higher scores indicating better functioning and quality-of-life.

The Clinician Global Impression of Severity (CGIS) [28] is a single-item rating that asks the clinician to evaluate the severity of the patient's GAD symptoms on a 7-point scale (1 = "Not at all ill" to 7 = "Among the most extremely ill patients"): "Considering your total clinical experience, how severe are the patient's symptoms now, compared to your experience with other patients with the same diagnosis?" The Clinician Global Impression of Change (CGIC) and Patient Global Impression of Change (PGIC) are two additional items that address change in the severity of a patient's illness over a particular time interval, in the present context "since the start of the study." The CGIC, like the CGIS, is completed by the clinician, whereas the PGIC is patient-reported. Both items employ a 7-point response scale (1 = "Very Much Improved" to 4 = "No Change" to 7 = "Very Much Worse").

#### Statistical Methods

##### Reliability

At-home test-retest reliabilities were computed using stable patients whose HAM-A change scores from screening to baseline (randomization) was 1 point or less. Data from Day -6 were used as the initial or "test" administration and Day -5 as the "retest" administration; reliabilities were also calculated for Day -5 to -4, Day -4 to -3, Day -3 to -2, and Day -2 to -1. Intraclass correlation coefficients (ICCs) were computed using a two-way (subjects × time) random effects analysis of variance (ANOVA) model as recommended by Schuck [29] and Shrout and Fleiss [30].

##### Responsiveness

For utility in clinical trials, it is important that the GA-VAS be capable of detecting change over time, preferably at more than one time-point to understand the onset and durability of the effect. Guyatt's responsiveness statistic [31] is an effect size estimate recommended for use in the evaluation of responsiveness. We calculated Guyatt's statistics at Weeks 1, 2, and 4 in order to compare three different types of HAM-A responders to non-responders. Initial responders were defined as those patients who achieved ≥ 50% reduction in HAM-A scores at Week 1, regardless of their responder status at Weeks 2 and 4; partial responders were patients who achieved ≥ 30% reduction in HAM-A scores at Week 1 (again, regardless of responder status at Weeks 2 and 4); sustained responders were patients who achieved ≥ 30% reduction in HAM-A scores at Weeks 1 and 2, and ≥ 50% reduction in HAM-A scores at Week 4. It was anticipated that Week 1 responsiveness statistics comparing initial responders and non-responders would be greater than responsiveness statistics comparing partial responders and non-responders or sustained responders and non-responders, with the responsiveness statistics based on the latter two comparisons being very similar at Week 1. To the extent that GAD symptoms return at Weeks 2 and 4 in initial and partial responders, it was expected that those responsiveness statistics would become smaller in size. It was further expected that the Guyatt's statistics involving sustained responders and non-responders would maintain a high level of responsiveness over all three time-points.

Computing change as the difference between Day 1 (baseline) and Week 1 (or Week 2 or Week 4), we calculated Guyatt's responsiveness statistics [31] for the three different responder definitions at three time-points:(1)

The resulting value is a measure of the effect of treatment on GAD symptoms. Cohen [32] provides a general rule-of-thumb for the interpretation of such effect size estimates: effect sizes of about 0.20 represent small effects, those of about 0.50 represent moderate effects, and those greater than about 0.80 represent large effects.

It is also important to demonstrate that the GA-VAS is sensitive to differences between treatment groups. We computed Cohen's [32] effect size estimate at Weeks 1, 2, and 4 in order to compare each active treatment to placebo: (Mean_Treatment _- Mean_Placebo_)/SD_Pooled_

##### Construct Validity

Construct validity describes the relationships among multiple indicators of a construct and the degree to which they follow predictable patterns [33]. Correlations between the GA-VAS and the HAM-A, HADS, QLES-Q, SF-36, and CGIS were computed using data collected during clinic visits at screening, baseline, and Weeks 1, 2, and 4. It was expected that the GA-VAS would correlate relatively highly with the other measures of anxiety—the HAM-A, HADS-Anxiety, and CGIS. As evidence for divergent validity, it was also anticipated that the GA-VAS would correlate more highly with the HADS-Anxiety score than with HADS-Depression and also more highly with the QLES-Q and the mental functioning subscales of the SF-36 (i.e., Emotional Role, Mental Health, Social Function, Vitality) compared to the SF-36 physical functioning subscales (i.e., Physical Function, Physical Role, Bodily Pain, General Health).

##### Minimum Important Differences (MIDs)

Another useful property of an outcome measure is the MID or the smallest change in a score from baseline that patients perceive as beneficial and would be clinically significant. Several methods have been proposed to assess clinically meaningful change, for example, patient- and physician-based global judgments and statistical criteria. One relatively common approach is to examine the distribution of change scores on a measure in conjunction with patients' global ratings of change [34]. In the present analysis, both PGIC and CGIC data were used as anchors to produce MID estimates. A simple MID estimate is taken to be roughly equivalent to the mean GA-VAS change of patients who reported they were "Minimally Improved."

The standard error of measurement (SEM), as recommended by Wyrwich et al. [35], for the GA-VAS was also computed:  where *SD *is the standard deviation of the subscale score and *r *is the test-retest reliability estimate. This is a distribution-based MID estimate that also considers measurement precision and has been shown to be relatively stable across populations [36]. We also explored the use of a 0.5 standard deviation (half-*SD*) unit change in the GA-VAS [37] as a final estimate of MID.

## Results

### Preliminary Qualitative Study

The cognitive interviews conducted with GAD patients showed that the GA-VAS was well understood and easily completed by all interview participants. Subjects raised no concerns about averaging anxiety levels over the last 24 hours. Overall, participants generally felt that the single-item GA-VAS was useful and could adequately capture the overarching GAD construct.

### Psychometric Study

A total of 167 GAD patients participated in the study; 97 (58.1%) were female and 122 (73.1%) were white. Table 1 summarizes patient characteristics by treatment group. At screening, patients averaged 67.26 (SD = 16.1) on the GA-VAS scale, and 62.61 (SD = 19.9) at baseline. Average GA-VAS scores declined (i.e., improved) with treatment: 49.16 (SD = 23.8) at Week 1, 43.35 (SD = 25.0) at Week 2, and 35.76 (SD = 24.8) at Week 4.

**Table 1:** Patient characteristics (Intent-to-treat population)

	Treatment Arm		
	Placebo (*n *= 57)	Paroxetine (*n *= 55)	Lorazepam (*n *= 55)
Gender (*n*, %)			
Male	26, 45.6%	24, 43.6%	20, 36.4%
Female	31, 54.4%	31, 56.4%	35, 63.6%

Race (*n*, %)			
White	42, 73.7%	40, 72.7%	40, 72.7%
Black	3, 5.3%	3, 5.5%	3, 5.5%
Hispanic	9, 15.8%	6, 10.9%	8, 14.4%
Other	3, 5.3%	6, 10.9%	4, 7.2%

Age in years (mean, *SD*)	35.0, 10.4	34.7, 12.6	38.5, 12.1
Baseline HAM-A (mean, *SD*)	24.2, 5.0	23.4, 3.3	24.2, 3.5

#### Reliability

The at-home GA-VAS test-retest stabilities were found to be adequate for a single-item measure: Day -6 to -5, 0.59; Day -5 to -4, 0.61; Day -4 to -3, 0.50; Day -3 to -2, 0.60; and Day -2 to -1, 0.52.

#### Responsiveness

Table 2 presents the results of the responsiveness analyses, which indicate highly satisfactory levels of responsiveness for the GA-VAS using all three responder definitions for comparing responders vs. non-responders across all time-points. The Guyatt's statistics are moderate to large in magnitude—all statistics exceed 0.70. GA-VAS responsiveness for initial responders diminished from -1.13 at Week 1 to -0.71 at Week 2 and -0.79 at Week 4, as expected. Responsiveness statistics based on partial and sustained responders were smaller at Week 1 than that for initial responders; however, there was very little attenuation of the responsiveness for either partial responders or sustained responders over time.

**Table 2:** Responsiveness of the GA-VAS at Weeks 1, 2, and 4 (In-clinic Visits)

	Week 1	Week 2	Week 4
**Guyatt's Responsiveness Statistics**			
Initial responder (n = 121) vs. Non-responder (n = 19)	-1.13	-0.71	-0.79
Partial responder (n = 90) vs. Non-responder (n = 50)	-0.92	-0.89	-0.86
Sustained responder (n = 79) vs. Non-responder (n = 32)	-0.91	-0.89	-0.80
**Cohen's Effect Size Estimates**			
Placebo (n = 54) vs. Lorazepam (n = 46)	0.42	0.49	0.29
Placebo (n = 54) vs. Paroxetine (n = 48)	-0.07	0.59	0.67

*Note: *Initial responders achieved ≥ 50% reduction in HAM-A scores at Week 1 (regardless of responder status at Weeks 2 and 4); partial responders achieved ≥ 30% reduction in HAM-A scores at Week 1 (regardless of responder status at Weeks 2 and 4); sustained responders achieved ≥ 30% reduction in HAM-A scores at Weeks 1 and 2, and ≥ 50% reduction in HAM-A scores at Week 4. Initial, partial, and sustained responder categories were not mutually exclusive.

The Cohen's effect size estimates for the treatment group comparisons are somewhat smaller in size, but still acceptable. While the Placebo vs. Lorazepam comparisons yield statistics with positive signs because Lorazepam reduces anxiety better than Placebo, the comparisons involving Placebo and Paroxetine subjects show that the groups are initially similar (-0.07 at Week 1), but by Week 2 Paroxetine subjects score lower (better) on the GA-VAS than Placebo subjects (0.59).

#### Construct Validity

Correlations between the GA-VAS and other available measures were computed using data collected during clinic visits (screening, baseline, and Weeks 1, 2, and 4) and are displayed in Table 3. The correlations are generally smaller at screening and baseline and increase at later time-points with treatment. As anticipated, the GA-VAS correlated highly with other measures of anxiety (the HAM-A, HADS-Anxiety, and CGIS), demonstrating convergent validity. With respect to divergent validity, it was hypothesized that the GA-VAS would correlate more highly with the HADS-Anxiety than with the HADS-Depression; this was found to be true. As expected, the GA-VAS correlated negatively with the QLES-Q and the SF-36 subscales, indicating that greater anxiety was associated with poorer functioning and quality of life. Also as hypothesized, larger correlations were obtained between the GA-VAS and the mental subscales of the SF-36 compared to the physical subscales.

**Table 3:** Correlations Between the GA-VAS and Other In-Clinic Measures

	Screening	Baseline	Week 1	Week 2	Week 4
HAM-A	0.31	0.31	0.53	0.61	0.60
HADS - Anxiety	0.47	0.40	0.63		0.74
HADS - Depression	0.26	0.26	0.44		0.63
CGIS	0.27	0.26	0.49		0.63
QLES-Q		-0.31	-0.45	-0.55	-0.54
SF-36 Emotional Role		-0.28	-0.45		-0.54
SF-36 Mental Health		-0.32	-0.57		-0.68
SF-36 Vitality		-0.27	-0.34		-0.49
SF-36 Social Function		-0.22	-0.40		-0.48
SF-36 General Health		-0.30	-0.30		-0.31
SF-36 Physical Function		-0.19	-0.21		-0.24
SF-36 Physical Role		-0.15	-0.23		-0.31
SF-36 Bodily Pain		-0.24	-0.16		-0.26

#### MIDs

The distributions of GA-VAS scores for each of the seven PGIC response categories are presented in Table 4. A simple MID estimate is taken to be roughly equivalent to the mean GA-VAS change of the "Minimally Improved" patients—in the PGIC analyses, approximately 13.5 to 15.5 GA-VAS points; using the CGIC, the MID estimate is about 26.6 GA-VAS points. For comparative purposes, the PGIC-based HAM-A MID was computed to be 7.40 and the CGIC-based HAM-A MID was computed to be 8.12. The half-*SD *GA-VAS MID estimates are slightly smaller than the PGIC- and CGIC-based estimates: 9.96 at baseline, 11.9 at Week 1, 12.5 at Week 2, and 12.39 at Week 4. The SEM-based MID estimate is 2.82, quite a bit smaller than the other MID estimates. Overall, the GA-VAS MIDs range in size from 2.8 to 26.6, but cluster between 10 and 15. A preliminary workable MID value for the GA-VAS is approximately 12 or 13 on the 100-point GA-VAS scale.

**Table 4:** MIDs - Distribution of Mean Change Scores for the GA-VAS

	PGIC - Mean Change at Week 1 (In-clinic)	PGIC - Mean Change at Week 4 (In-clinic)	CGIC - Mean Change at Week 4 (In-clinic)
1 = "Very Much Improved"	38.50 *n *= 6	45.63 *n *= 16	41.78 *n *= 18
2 = "Much Improved"	28.97 *n *= 29	34.57 *n *= 44	30.55 *n *= 31
**3 = "Minimally Improved"**	**13.45 *n *= 58**	**15.54 *n *= 35**	**26.61 *n *= 43**
4 = "No Change"	1.56 *n *= 36	5.00 *n *= 12	4.58 *n *= 19
5 = "Minimally Worse"	2.00 *n *= 9	9.67 *n *= 3	47.00 *n *= 1
6 = "Much Worse"	6.25 *n *= 4	28.50 *n *= 4	*n *= 0
7 = "Very Much Worse"	*n *= 0	*n *= 0	*n *= 0

## Discussion

We have evaluated a patient-reported VAS for use in assessing onset of improvement in anxiety symptoms in subjects with GAD sooner than one week. The qualitative results demonstrated that GAD patients had no difficulties with the GA-VAS format or reporting average anxiety levels over the last 24 hours, which has been shown to be more reliable than asking for a rating at a specific point in time [38].

A set of analyses was aimed at providing evidence of the reliability, responsiveness, validity, and utility of the GA-VAS. The GA-VAS demonstrated marginally adequate test-retest stability. Based on similar reliability results using other measures that were administered daily in this study, it is likely that patients were not stable during the screening period, but were experiencing small changes in GAD symptoms which affected the reliability of the GA-VAS. The present reliabilities were somewhat lower than what has been reported for other domains and outcomes, such as pain [39,40]. However, it is difficult to compare these reliabilities to findings for other patient-reported VASs because other VASs use different time intervals (e.g., 5 minutes, one week), experiential dimensions (e.g., current pain, average pain, worst pain), and different statistical methods (e.g., Pearson correlations).

Three different definitions of responder were used in this analysis, all based on changes in the clinician-rated HAM-A. All Guyatt's statistics show the GA-VAS to be highly responsive—changes in GA-VAS scores at Weeks 1, 2, and 4 in subjects classified as responders exceeded changes of non-responders. The comparison of initial responders vs. non-responders at Week 1 produced the largest responsiveness statistic, but responsiveness for these initial responders declined at Weeks 2 and 4, while responsiveness for partial responders and sustained responders remained relatively steady over time. The Cohen's effect size estimates were mostly moderate in size, but corroborate the responsiveness of the GA-VAS.

Validity correlations between the GA-VAS and other available measures were highly satisfactory. Specifically, the GA-VAS obtained relatively high correlations with the HAM-A, HADS-Anxiety, and the mental subscales of the SF-36, and lower correlations with the HADS-Depression and the physical subscales of the SF-36. At Weeks 1, 2, and 4, all observed correlations fit the hypothesized pattern of relationships, except that the correlations between the GA-VAS and HADS-Depression scores were possibly greater than expected.

As noted, the correlations between the GA-VAS and other measures are smaller at screening and baseline and increase at later time-points with treatment (Table 3). This is particularly true for the psychological measures (HAM-A, HADS-Anxiety, HADS-Depression, and CGIS) and psychosocial functional status measures (QLES-Q and SF-36 Emotional Role, Mental Health, Social Function, Vitality). This is probably due to the increasing variability in both the GA-VAS and these other measures—those patients who responded to treatment achieved better scores on measures of anxiety and psychosocial functioning, which increased the overall variability in these measures and reduced the relatively restricted range present at screening and baseline.

For exploratory purposes, three different MID estimates were computed, and the results vary across methods but seem plausible. Inconsistencies among MID estimates computed using multiple methods is to be expected [41,42], and PGIC- and CGIC-based MIDs are not necessarily expected to be consistent because the clinician perspective naturally differs somewhat from that of the patient. The results point toward a preliminary MID value of approximately 12 or 13 points on the 100-point GA-VAS scale.

The present findings are preliminary and the psychometric characteristics of the GA-VAS should be confirmed in future studies. This analysis was conducted as part of a rigorously controlled clinical trial and the results are applicable in a clinical trial setting—how the GA-VAS will perform in other settings is unknown. Furthermore, the present results are based on limited psychopharmacological agents, and exclude comparisons with important cognitive-behavioral interventions and alternative therapies.

## Conclusions

The present study demonstrates the reliability, validity, and responsiveness of the GA-VAS measure in the context of daily administration in diary format, as well as in-clinic administration. The GA-VAS successfully minimizes patient burden while capturing early onset of medication action and symptom relief. With these advantages in mind, we recommend use of the GA-VAS in future research studies and clinical trials for the evaluation of fast-acting drug therapies for the treatment of GAD.

## Competing interests

VSLW and RJM declare that they have no competing interests. DF is an employee of Pfizer Inc.

## Authors' contributions

VSLW conducted the psychometric analyses and drafted major portions of the manuscript. RJM and DF conceived of the study and participated in its design and analysis, and drafted major portions of the manuscript. All authors read and approved the final manuscript.
